# Immunomodulatory effects of icariin in a myocardial infarction mouse model

**DOI:** 10.1080/21655979.2022.2076453

**Published:** 2022-05-17

**Authors:** Xiyalatu Sai, Zhetao Li, Gang Deng, Lu Wang, Wang Xiaowu, Moussa Ide Nasser, Chi Liu, Ping Zhu

**Affiliations:** a The Second School of Clinical Medicine, Southern Medical University, Guangzhou, China; b Guangdong Cardiovascular Institute, Guangdong Provincial People’s Hospital, Guangdong Academy of Medical Sciences, Guangzhou, China; c Affiliated Hospital of Inner Mongolia University for the Nationalities, Tongliao City, People's Republic of China; d Department of Nephrology, Sichuan Academy of Medical Science and Sichuan Provincial People’s Hospital, Sichuan Renal Disease Clinical Research Center, University of Electronic Science and Technology of China, Chengdu, China; e Chinese Academy of Sciences Sichuan Translational Medicine Research Hospital, Chengdu, China

**Keywords:** Icariin, nrf2/HO-1, myocardial infarction, inflammatory, immune response

## Abstract

Myocardial infarction (MI) is a prevalent cardiovascular disease defined by myocardial ischemia and hypoxic damage caused by plaque rupture, thrombosis, lumen stenosis, or blockage in the coronary artery. However, the development of emergency percutaneous coronary interventional therapy has enabled the rapid restoration of blood perfusion to ischemic myocardium and the rescue of dying myocardium cells. Some dying myocardium cells have caused irreversible damage and impaired cardiac function recovery in recent years. Icariin has been utilized to treat various ailments as a natural chemical extract. In this study, we employed a variety of approaches to observe MI, including western blotting, quantitative RT–PCR, immunohistochemistry, and flow cytometric analysis using icariin. As demonstrated by the research findings, icariin may prevent MI-induced cell apoptosis. This is accomplished by inhibiting proinflammatory factors via the Nrf2/HO-1 signaling pathways. These data imply that icariin may be an effective treatment for MI.

## graphical abstract

**Figure:**
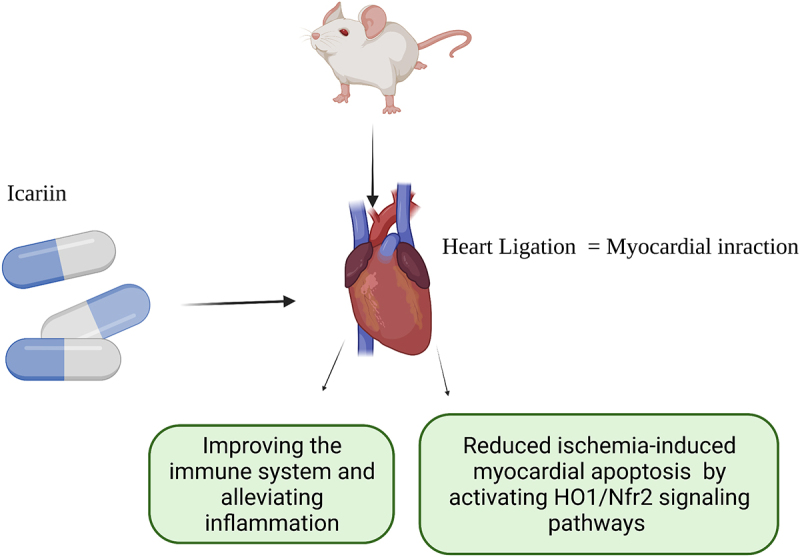

## Highlights

- Icariin prevents Myocardial infraction by improving the immune system
- Icariin treatment reduced ischemia-induced myocardial apoptosis
- Icariin prevent Myocardial infraction by HO-1/Nrf2

## Introduction

Myocardial infarction (MI) is a condition in which the coronary artery is chronically ischemic and hypoxic, resulting in myocardial ischemic necrosis. MI is a primary cause of death in humans and can be aggravated by malignant arrhythmia, cardiac failure, or shock [1]. MI is associated with a high rate of morbidity and mortality in the majority of developed countries and has developed into a substantial public health issue [2]. Following myocardial infarction, oxidative stress is a primary cause of myocardial cell death, and myocardial cell apoptosis contributes to the incidence and development of MI [3]. Similarly, apoptosis occurs more often in myocardial cells during MI, and cardiac remodeling ultimately culminates in heart failure. As a result, decreasing oxidative stress and reversing myocardial apoptosis following MI are key components of MI therapy [4].

Numerous studies on the immunology of cardiovascular diseases have revealed a close link. T lymphocytes are the key regulators of the immune system in the body, and the role of dysregulated regulatory T (Treg) cells in cardiovascular illnesses has recently garnered more attention [5,6]. Recently, the effect of Tregs in improving healing after myocardial infarction by modulating monocyte/macrophage polarization has been reported. Following myocardial infarction, Treg depletion results in decreased cardiac function, massive infiltration of inflammatory cells, and sparse collagen deposition in the scar.

As an injury-associated protein, heme oxygenase (HO-1) suppresses the activation of inflammatory cells, such as lymphocytes, macrophages, synovial fibroblasts, and dendritic cells. Inhibition of HO-1 expression results in immunosuppression of Treg cells, which are critical for controlling cardiovascular disease. Nrf2 forms a signaling pathway with the antioxidant protein HO-1 [7,8]. Modern medicine has made no substantial advancements in the treatment of MI thus far. Currently, MI is mainly treated with medication, immune cell transfer, and other procedures. Nonetheless, these therapies have several disadvantages, including variable efficacy, considerable side effects, and a high cost. As a result, minimizing myocardial damage caused by MI has been an important research topic in cardiovascular disease medicine. Developing effective treatment agents to treat MI is critical [9,10].

Icariin is a traditional Chinese medicine extracted from medicinal plants. Herba Epimedii has demonstrated numerous pharmacological actions, including aphrodisiac, synaptic formation, protecting neurons from damage, boosting bone metabolism, and possessing anti-inflammatory, antioxidative stress, antidepressive, and antidepressive antitumor properties [11–13]. Icariin has been widely examined over the last few years and has been shown to have antioxidant, anti-inflammatory, and anti-apoptotic properties. It is presently being investigated as a possible treatment for a multitude of diseases, ranging from cancer to cardiovascular disease [14]. Likewise, Icariin has been proven in previous investigations to preserve myocardium function in rats following myocardial I/R damage. Indeed, icariin has been shown to minimize IS, I/R damage, and intestinal remodeling. These properties of icariin-associated blood indicator concentrations of CK, IMA, and serum LDH and activation of the Nosh/PI3K/Akt pathway in ischemic tissue make icariin a plausible option for preventing and repelling I/R damage in the early stages of the disease [15].

Additionally, icariin has been shown to protect the cardiovascular system by increasing the expression of Nrf2 and HO-1, which regulate lipid levels and protect against atherosclerosis in various ways, including reducing oxidative stress and lowering chemokines [16]. Icariin may also enhance testicular tissue’s antioxidant capacity by activating the Nrf2/HO-1 signaling pathway, protecting testicular germ cells from DNA damage in typically aged rats, and maintaining rat reproductive function [17]. Similarly, icariin may have anti-inflammatory actions, inhibit NF-κB activation and the synthesis of downstream inflammatory factors, decrease NADPH oxidase activity in animals, and protect against H_2_O_2_-induced oxidative stress [18]. Similarly, icariin lowered the total white blood cell count in lung failure mouse models and regulated the Th17/Treg balance [19]. Although numerous studies indicate that icariin is likely to influence immune system function through the Nrf2/HO-1 pathway, no reports corroborate these ideas. However, the function of icariin in MI and its related molecular mechanisms have not been investigated. Therefore, we hypothesized that icariin might be a promising candidate for MI treatment. This study demonstrated that icariin acts as an effective agent against MI model mice in vivo. The Nrf2/HO-1 pathway is likely to be involved in increased icariin-mediated Treg cells. Our findings suggest that icariin could serve as a potential agent for the treatment of MI.

## Materials and methods

### Drugs & chemicals

Icariin was purchased from MEYER company (Shanghai, China), whereas the antibodies used for western blotting were purchased from Cell Signaling Technology (Danvers, MA). The antibodies were anti-CD3 mAb (PE), anti-CD4 mAb (APC), anti-CD8a mAb (PE/Cy7), anti-CD25 mAb (APC/Cy7), anti-CD44 mAb (FITC), anti-CD62L mAb (APC/Cy7), anti-IFN-γ mAb (PE), HO-1 mAb (PE/Cy7), anti-CD16/32 mAb and anti-Foxp3 mAb (FITC) (eBioscience, San Jose, CA, USA).

### Animal models

Male mice (C57BL/6) weighing 22–28 g were procured from Hunan SJA Laboratory Animal Co., Ltd. They were maintained with the required alimentation and water in the animal house of Guangzhou Huateng Biomedical Technology Co., Ltd. All experiments were performed after institutional animal ethical committee approval 2019482A(R2).

### Experimental design

The mice were divided into three groups (sham, MI, and icariin) with each group with eight mice, and each experiment was replicated 3 times; they were acclimatized to a temperature of 28°C and a 12-hour light schedule. The mice were fed commercial pelleted mice food and water daily. Icariin (60 mg/kg) was administered orally for 28 days following dissolving in 0.5% DMSO (as a single dose/day). The mice in group I was given normal saline, group II represented the MI model, and group III was given Icariin.

### Echocardiographic measurements of cardiac function

At 28 days after ligation, the mice were anesthetized with sodium pentobarbital (50 mg/kg, i.p., Merck). We performed transthoracic echocardiography using an ultrasound machine (Vivid 7, GE Medical, Horten, Norway) equipped with a 10-MHz phased array transducer to test the left ventricular ejection fraction (EF) and calculated fractional shortening from M- (FS) [20].

### HE staining

The cardiac tissue was preserved in a 4% paraformaldehyde solution. Following alcohol dehydration, paraffin was embedded and sectioned to a thickness of 4 μm. Paraffin sections were cut, dewaxed with xylene, washed with ethanol, stained for 10 minutes with hematoxylin staining solution, and then stained for 2 min with eosin staining solution after ethanol washing. After dehydration, transparency, and sealing, the staining findings were examined under a microscope [21].

### Masson staining

To assess collagen deposition in different organs, cardiac tissue was stained with Masson. Sections were deparaffinized and washed in distilled water for 5 min, and nuclei were stained in Mayer’s hematoxylin (MUTO PURE CHEMICALS, Japan) for 5 min and washed in distilled water. Then, 0.8% Orange G solution (Wako, Japan) was applied to the slides for 2 min and rinsed in 1% acetic acid. Sections were then stained with azophloxin (Wako, Japan) for 20 min and rinsed in 1% acetic acid. A blue aniline solution (Wako, Japan) was applied to the slides for 1 min. A digital camera for light microscopy analysis was used to assess overall cellular and organ damage (BX51, OLYMPUS, Tokyo, Japan) and collagen deposition (stained blue) by morphometric analysis (MetaMorph software; Universal imaging, Downtown, PA) to quantify [22].

### Real-time quantitative RT–PCR

Total RNA was extracted from tissues or serum using TRIzol Reagent (TRIzol, Inc.). We electrophoresed a small amount of RNA to verify its integrity and then used ultraviolet (UV) spectrophotometry to measure the concentration and purity of the RNA. The solution was stored at −70°C until needed, and the concentration was adjusted as appropriate. Synthesis of the first strand of cDNA: Using the First Strand cDNA Synthesis Kit conversion reaction system, we added the sample to the PCR amplification equipment and ran the PCR amplification. Until cDNA’s reverse transcription product was obtained, the reverse transcription procedure described in the kit was followed until the end of the step. Q-PCR: The reaction system was designed and amplified using fluorescent quantitative PCR equipment, and the dissolution curve was plotted after each cycle was completed. The Ct value, which is the number of amplification cycles needed to produce a fluorescence signal above a given threshold for the amplified product during PCR amplification after the process was completed, was then established. Findings: The relative abundance of each gene was calculated using the (2-^∆∆CT^) method. Each experiment was replicated three times; the primers used are reported in Table 1.

**Table 1.** qPCR primer sequences

Genes	Primer Sequences (5’-3’)	Annealing temperature (°C)
*IL1β*	Forward: 5’- GAAATGCCACCTTTTGACAGTG-3 Reverse: 5’- TGGATGCTCTCATCAGGACAG-3	60
*HO-1*	Forward: 5’- AGGTACACATCCAAGCCGAGA-3 Reverse: 5’- CATCACCAGCTTAAAGCCTTCT-3	60
*IFN-γ*	Forward: 5’- GGCCATCAGCAACAACATAAG- −3 Reverse: 5’- TGGGTTGTTGACCTCAAACT −3’	60
*TNF-α*	Forward: 5’- GCCTCCCTCTCATCAGTTCTAT-3’ Reverse: 5’- CACTTGGTGGTTTGCTACGA −3’	60
*α-SMA*	Forward: 5’- CCCAGACATCAGGGAGTAATGG-3’ Reverse: 5’- TCTATCGGATACTTCAGCGTCA-3’	60
*18S*	Forward: 5’- CGCCGCTAGAGGTGAAATTC −3’ Reverse: 5’- CGAACCTCCGACTTTCGTTCT-3’	60

### Flow cytometry

The BD company’s flow cytometry was used to detect the lymphocyte subpopulation: various fluorescein-labeled monoclonal antibodies were added to whole blood, bound to the corresponding antigen on the lymphocyte membrane, and analyzed by flow cytometry following hemolysis, washing (and fixing), and other steps. The numbers of IFN-γ, Foxp3, and CD8 cells were determined using flow cytometry, and the number of Treg-related cells in each group was thoroughly examined [23].

### Western blotting

To further validate the protein expression levels of key molecules in cardiac tissues, the protein levels of HO-1, Nrf2, P38, P-P38, Bcl-2, Bax, cytochrome c, and cleaved caspase-3 were determined. The tissues were sliced into 1 mm^3^ piece, thoroughly washed with PBS, and treated with protein extract. Centrifugation, denaturation, use of the supernatant, and storage at −80°C were performed. The samples were mixed in equal volumes for 10 min and then separated by SDS–PAGE. Following electrophoresis, wet transfer electrophoresis was used to transfer the proteins to a PVDF membrane. The membranes were sealed and treated overnight with primary antibodies at 4°C. The membranes were treated for 2 h at room temperature with each secondary antibody from the mouse and rabbit. Following ECL color development, data for photography were collected using Bio–Rad imaging equipment [24].

### Immunofluorescence

Tissues were fixed with ice-cold 2% paraformaldehyde and permeabilized with 0.2% Triton X-100 (TX100). Fixed cells were incubated for 1 h in 1% bovine serum albumin (BSA), stained with primary antibodies for 1 h, and exposed to secondary antibodies for 45 min. Images were acquired using a Zeiss 710 confocal microscope and processed using ImageJ (NIH) and Photoshop CS5.1 software (Adobe).

### Immunohistochemical staining

To further validate the expression and distribution of critical molecules in the heart, IL-17 was identified in heart tissues. Immunohistochemical staining was performed by utilizing a Discovery XT, a Roche-manufactured automated, multifunctional histopathology detection system. The major procedures comprised standard paraffin section dewaxing and hydration, trypsin antigen repair, hydrogen peroxide to eliminate the endogenous peroxidase, a Triton membrane, and normal serum occlusion. Overnight at 4°C, a primary antibody was added. A biotinylated goat anti-mouse secondary antibody was added, and streptomycin labeled with horseradish enzyme was added. The color was developed using DAB, and the tablets were clear and sealed. Each field’s staining intensity was determined using Image-Pro Plus 7.0. Ten fields were randomly chosen from each specimen, and the mean value was used to reflect the staining intensity of a particular protein in animal tissue [25].

### Statistical analysis

Data were analyzed using GraphPad Prism 7.0 software (La Jolla, CA, USA). All statistical tests were applied as indicated, and p < 0.05 was considered significant. Data are plotted as the mean ± S.E.M.

## Results

Icariin has been shown to protect cardiomyocytes from oxidative stress by activating the ERK signaling pathway [26]. Similarly, icariin has been shown to decrease heart muscle collagen content and mitochondrial ROS levels in rats with hypoglycemic disease, boost SOD activity and improve cardiac function [27]. The molecular mechanism by which icariin induces cardioprotective effects in mice after MI has not been established. This research aims to demonstrate the cardioprotective impact of icariin after MI.

### Effect of icariin on MI

**Figure 1.**
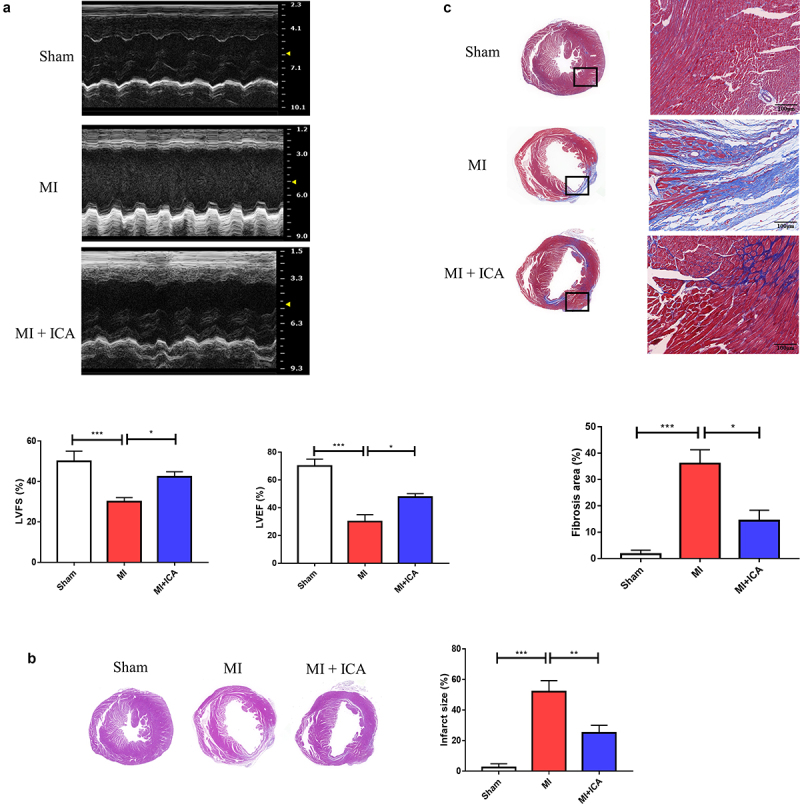
**Icariin improves cardiac function after myocardial infarction**: (a) Thoracic echocardiography of mice after 28 days of treatment compared to the sham group. (b) Illustration of H&E staining and measurement of the cross-sectional area of the fibers in the hearts (× 100). (c) Digitalized slides of Masson’s trichrome-stained heart paraffin slices and collagen volume. Collagen has been dyed in blue, and the cytoplasm has been stained in red, respectively (× 100).

Thoracic echocardiography was used to assess heart function four weeks after MI. Compared to the Sham group, the MI group’s LVEF, + DP/DTmax, and -DP/DTmax were considerably decreased; compared to the MI group, the icariin group’s LVEF, + DP/DTmax, and -DP/DTmax were dramatically increased (*P < 0.05). (Figure 1a).

Ischemia causes dysfunction in myocardial cells and causes alterations in the morphology of the cells in the heart. This research assessed pathological alterations using HE staining to determine if icariin provided cardioprotection. As shown in Figure 1b, cardiac cells in the model group suffered severe damage, and many neutrophils invaded the necrotic tissue. Compared to the sham group, aberrant distribution of the surviving myocardial cells was observed. Despite this, mice in the icariin-treated groups showed a substantial improvement in their condition.

Moreover, Masson’s trichrome staining technique was used to identify fibrosis deposits in the myocardium. The color of fibrous tissue was blue, while the color of cardiac fibers was red. When comparing the model group to the sham group, cardiac fibers’ collagen content and disarray were substantially higher in the model group. In Figure 1c, the ratios of fibrotic tissue to normal tissue are compared. Nevertheless, various doses of icariin therapy alleviated cardiac fibrosis to varying degrees; the collagen content was significantly reduced compared with that of the model group, and high-dose treatment resulted in greater therapeutic effectiveness.

### Icariin treatment reduced ischemia-induced myocardial apoptosis

**Figure 2.**
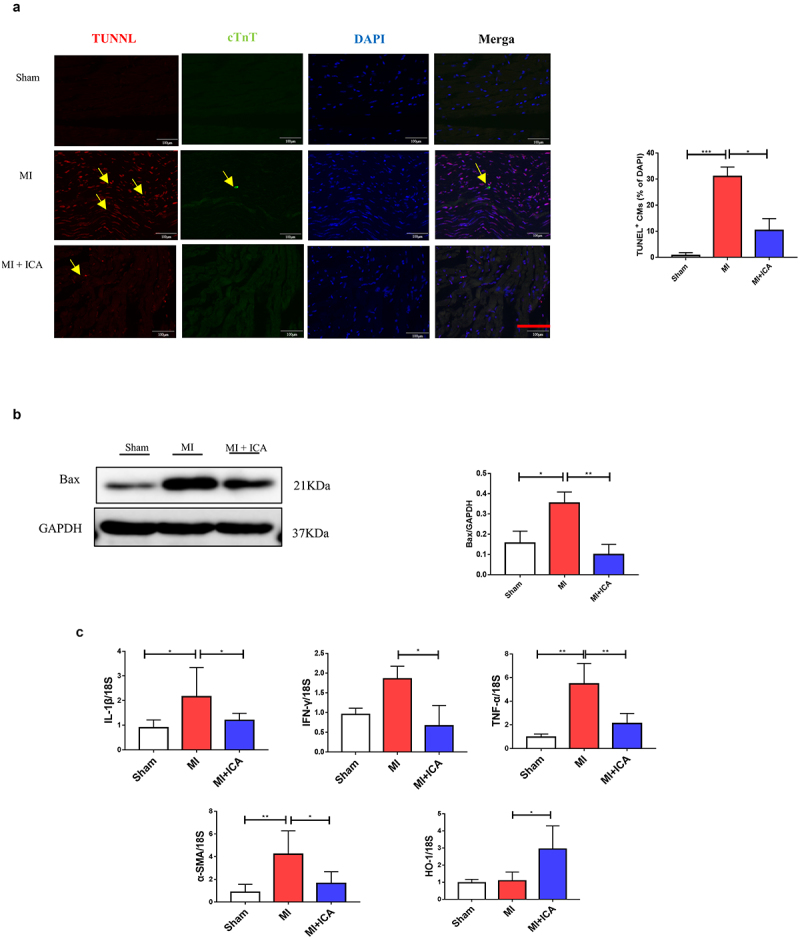
**Icariin prevents myocardial cell apoptosis**: (a) Mice myocardial were stained with TUNEL, *P < 0.05. (b) The protein expression of Bcl-2, Bax, cytochrome c, cleaved caspase-3, and GAPDH (control) were determined by Western blotting. Each experience was repeated 3 times *P < 0.05.

Next, the TUNEL test was used to assess myocardial apoptosis and protein expression 28 days after the ligation operations were performed (Figure 2). The nucleus was labeled blue with DAPI. Positive apoptotic cells were green. Because of the ischemia, there was more significant apoptosis in the model group than in the sham group. Additionally, the number of apoptotic mice in the model group was much greater than that in the sham group, which was surprising. Western blotting revealed that the proapoptotic factor Bax was downregulated compared to that in the sham group. Myocardial apoptosis and apoptosis were significantly decreased in the icariin treatment groups, and the expression of associated proteins was also changed.

### Icariin prevents MI by improving the immune system and alleviating inflammation

**Figure 3.**
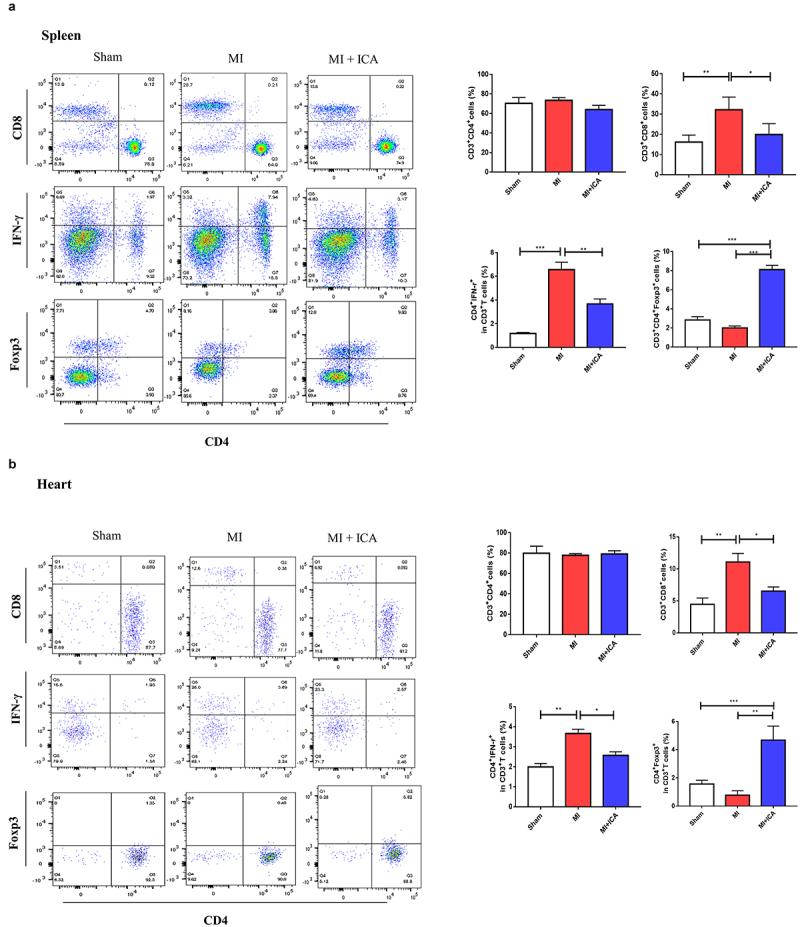
**Icariin improves mice immune system**: Flow cytometric analysis was performed to assess the population of lymphocytes (CD8+ CTL cells and CD3+ CD4++IFN-γ+ Th1) in the spleen and heart.

Immune dysfunction is the most frequent cause of MI. In clinical practice, T lymphocyte subsets may be used to express the immunological function of the body by expressing the immune function body, which is similar to the expression of the body’s immune function. It is possible to assess the equilibrium state of immune function by measuring the percentage of Foxp3+ and CD8^+^ cells present in the blood, both involved in regulating the immunological response. The lymphocyte population in the spleen and heart was analyzed using FCM. CD8+ CTL and CD3^+^CD4^+^IFN-γ^+^ Th1-cell populations were considerably lower in the ICA-treated group than in the sham group, but no significant change in CD3^+^CD4^+^ T-cell populations was identified between the two groups. Our results indicated that ICA-treated mice had a more significant proportion of CD3^+^CD4^+^Foxp3^+^ Treg cells than MI animals (Figure 3). Icariin alleviated inflammation by significantly decreasing the expression of TNF-α, IL-1β, INF-γ, and IL-17 (Figure 2c).

### Icariin prevents MI via HO-1/Nrf2

**Figure 4.**
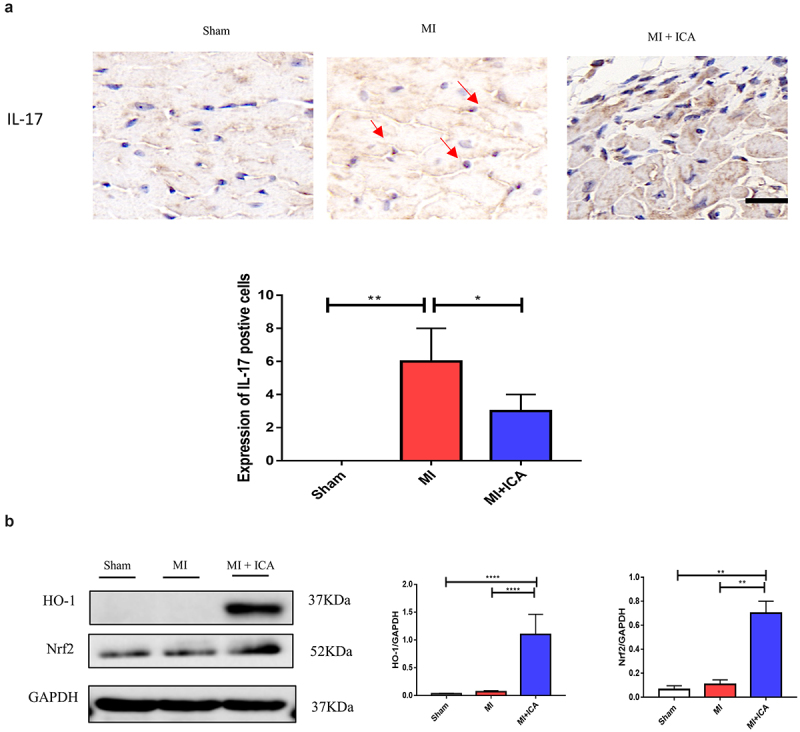
**Icariin promotes activation of Nrf2/HO-1 by alleviating inflammatory factors. Signaling pathways**: (a) Immunohistochemical was performed to check the expression of IL-17 (× 100); the experiment was repeated 3 times with means *P < 0.05. (b) qPCR was performed to evaluate the mRNA expression of IL1β, HO-1, TNF-α, and α-SMA compared to 18S. Each experience was repeated 3 times *P < 0.05. (c) The protein expression of HO-1, Nrf2, and GAPDH (control) was determined by Western blotting. Each experience was repeated 3 times *P < 0.05.

Many pathogenic variables control apoptosis of cardiac cells during MI, including ischemia, hypoxia, oxygen-free radicals, and inflammatory mediators. The Nrf2/HO-1 pathway is critical for myocardial ischemia and hypoxia regulation. Western blotting and qPCR were performed to evaluate the expression of Nrf2 and HO-1. As indicated in Figure 4, the expression of these proteins in the icariin group increased significantly, indicating that the Nrf2/HO-1 pathway is activated as a compensatory mechanism in infarcted myocardial tissue. Icariin may activate the Nrf2/HO-1 pathway further and serve as an anti-apoptotic and antioxidant.

## Discussion

An increase in permeability and oxygen free radicals induced by MI activate and engage various immunological responses, cytokines, and immune factors being created sequentially to contribute to cardiac inflammatory reactions and aggravate MI [28].

Recent evidence indicates that immunological abnormalities, including T-cell and body fluid immunity, may play a key role in the pathogenesis of MI [29,30]. Similarly, MI has been linked to reduced CD8^+^ T-cell immunological function. This means that immunological inflammation in patients with MI before initiating therapy may result in reduced immune function, deregulation of T lymphocyte subsets, immune system suppression, and damage, eventually increasing the instability of atherosclerotic plaques. A recent study revealed that CD8^+^ T lymphocyte activation contributes to the mechanism of coronary artery spasms. To be more precise, the damage caused to cardiac cells by activated CD8^+^ T lymphocytes has been used as a model for the development of MI; however, the specific mechanism is unknown [31,32]. Our work demonstrated that icariin suppressed CD8^+^ cells, indicating icariin’s significance in mouse function during MI.

TNF-α, IL-17, INF-γ, and IL-6 are frequently expressed inflammatory mediators in MI, and their levels correspond with the inflammatory state of ischemia-reperfusion damage. IL-17 is primarily produced by CD4^+^ T cells. These proinflammatory cytokines contribute to the process of tissue damage associated with autoimmune and inflammatory disorders, and their significance in MI is attracting attention [33,34]. Regardless, IL-17 is extensively expressed during myocardial ischemia-reperfusion damage, and exogenous antibodies have been shown to prevent MI considerably [29]. In this study, we used a mouse MI model and immunofluorescence to assess the expression of IL-17 in cardiac tissue. The expression of IL-17 was found to enhance MI, showing that IL-17 may have a role in MI.

Additionally, Treg cell deficiency is a major cause of MI. Previously published research has indicated that increasing HO-1 expression can induce Foxp3 expression, a critical marker of Treg cells [35,36]. We observed that icariin therapy might reduce inflammatory cytokine production and CD8^+^ cell activity, therefore ameliorating myocardial damage in MI mice. On this assumption, we recently focused on the immunomodulatory mechanism of icariin in MI via the HO-1/Nrf2 pathway and created an experimental design and research plan for this subject with a sound theoretical base.

The Nrf2/HO-1 signaling pathway is crucial for the in vivo promotion of the antioxidant activity. Massive generation of oxygen free radicals can activate Nrf2 transcription and function as an antioxidant via HO-1 activity. The Nrf2/HO-1 pathway is overexcited in cardiac tissue in an animal model of MI, and this overexcitation is considered one of the self-compensation formulae [37–39]. Following Icariin administration in MI mice, it was revealed that Nrf2 and HO-1 expression levels in cardiac tissue were significantly higher in the icariin group than in the MI group, suggesting that icariin can enhance antioxidant stress pathway activation during MI. As a result, the activated Nrf2/HO-1 pathway is proposed as a sub mechanism for icariin antioxidant action during MI.

## Conclusion

**Figure 5.**
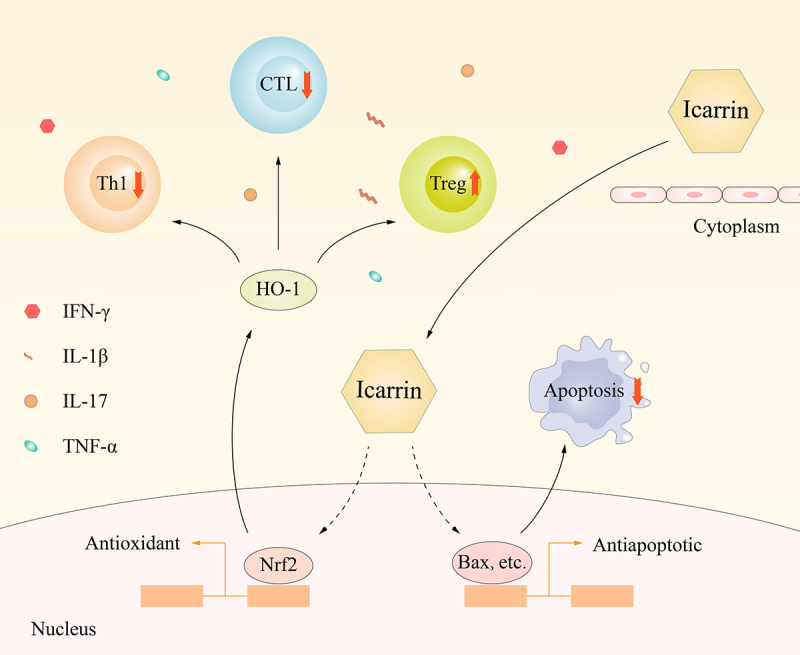
Molecular mechanism by which Icariin induces myocardial cell protection.

Icariin can minimize myocardial cell damage and the immune response in mice after acute myocardial infarction by alleviating inflammatory factors, and it can also activate the Nrf2/HO1 pathway (Figure 5).

## Supplementary Material

Supplemental Material
