# Supplementary material for: Immunomodulatory effects of icariin in a myocardial infarction mouse model
Source: Bioengineered. 2022 May 17;13(5):12504–15. doi: 10.1080/21655979.2022.2076453 (PMC9276034; doi:10.1080/21655979.2022.2076453)
Supplement: Supplemental Material [file KBIE_A_2076453_SM5677.zip › supplementary/certificate.pdf]

Editing Certificate

This document certifies that the manuscript

Immunomodulatory effects of Icariin in myocardial infarction mouse model

prepared by the authors

Xiyalatu Sai1, 2, 5 #, Zhetao Li1,2#, Gang Deng2 , Lu Wang2, Wang Xiaowu2, Moussa Ide Nasser2*, Chi Liu2, 3, 4* and Ping Zhu1, 2*

was edited for proper English language, grammar, punctuation, spelling, and overall style by one or more of the highly qualified native English speaking editors at AJE.

This certificate was issued on **April 25, 2022** and may be verified on the AJE website using the verification code **2AC5-4DFA-D770-D56B-046E**.

Neither the research content nor the authors' intentions were altered in any way during the editing process. Documents receiving this certification should be English-ready for publication; however, the author has the ability to accept or reject our suggestions and changes. To verify the final AJE edited version, please visit our verification page at aje.com/certificate. If you have any questions or concerns about this edited document, please contact AJE at support@aje.com.

AJE provides a range of editing, translation, and manuscript services for researchers and publishers around the world. For more information about our company, services, and partner discounts, please visit aje.com.
