# Supplementary figures and images for: Immunomodulatory effects of icariin in a myocardial infarction mouse model
Source: Bioengineered. 2022 May 17;13(5):12504–15. doi: 10.1080/21655979.2022.2076453 (PMC9276034; doi:10.1080/21655979.2022.2076453)

## Slide 1
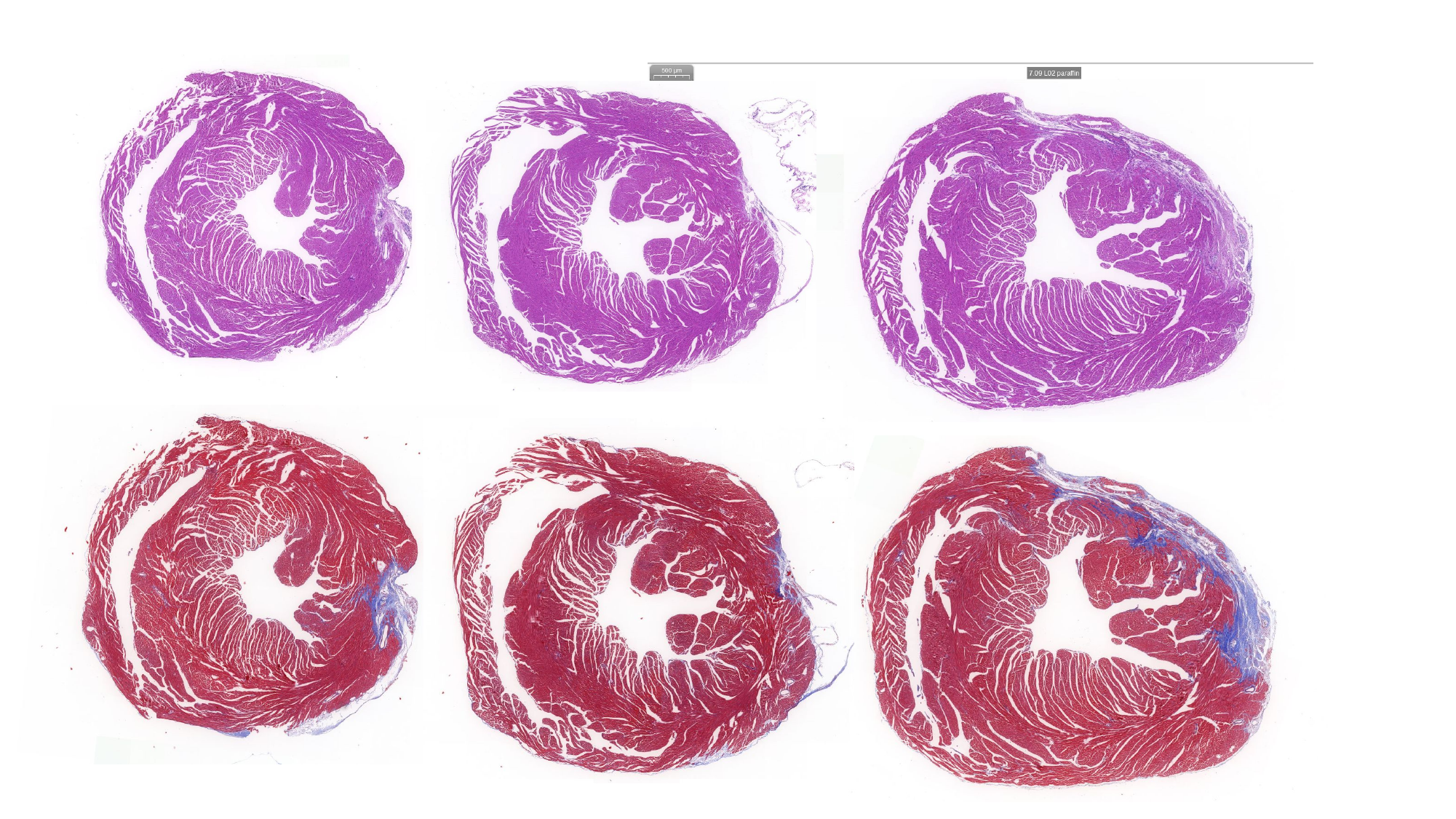

## Slide 2
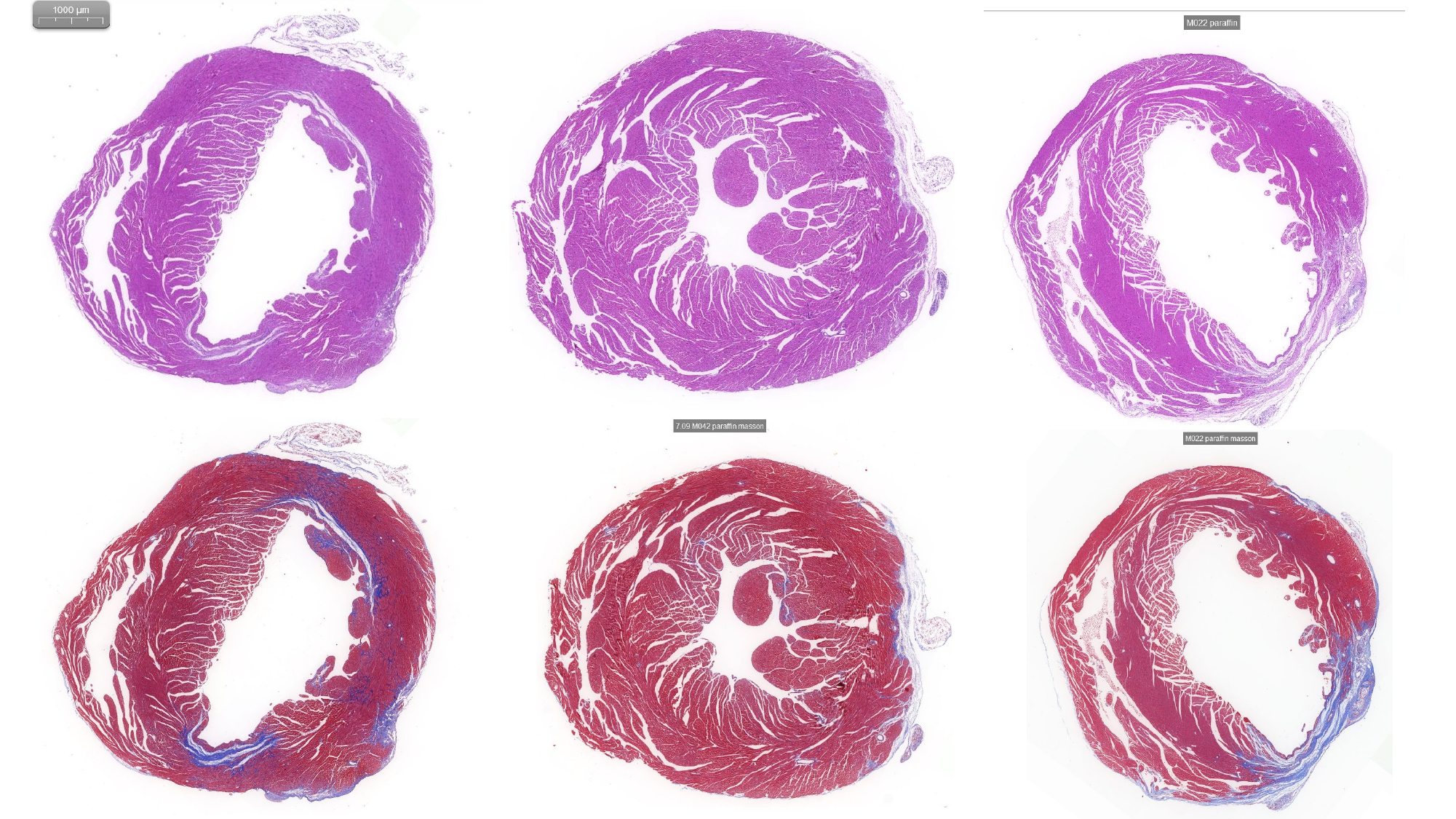

## Slide 3
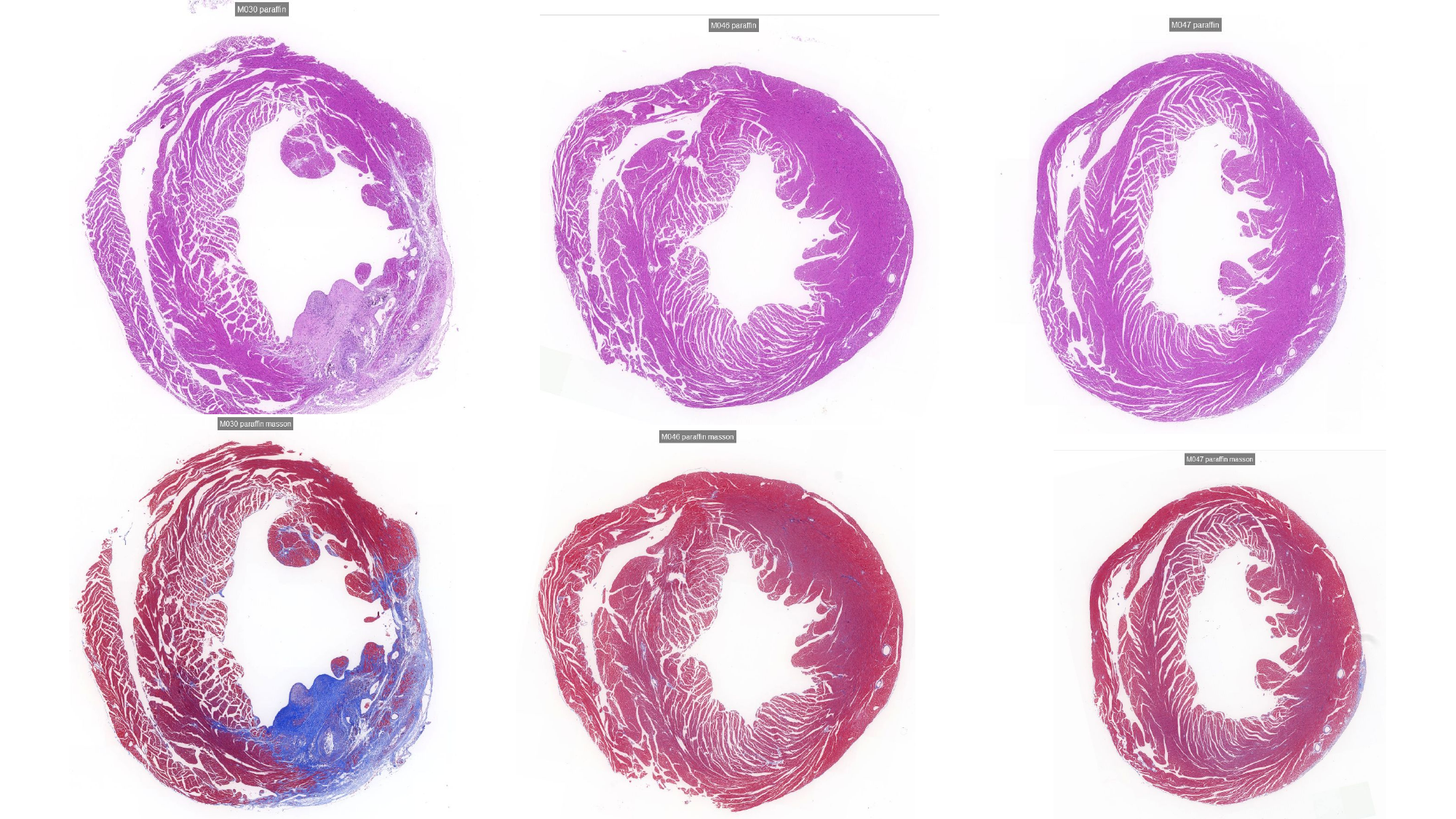

## Slide 4
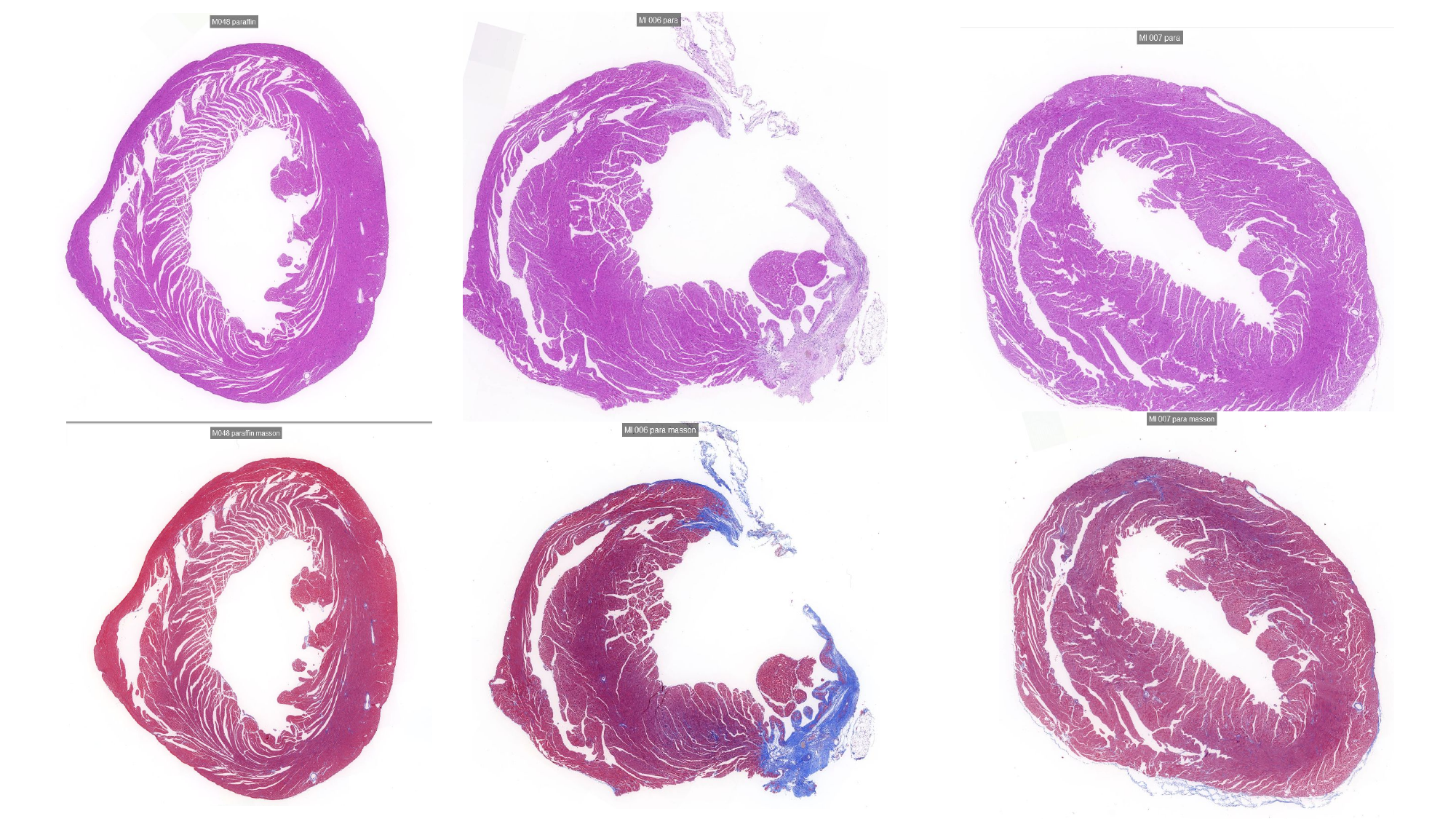

## Slide 5
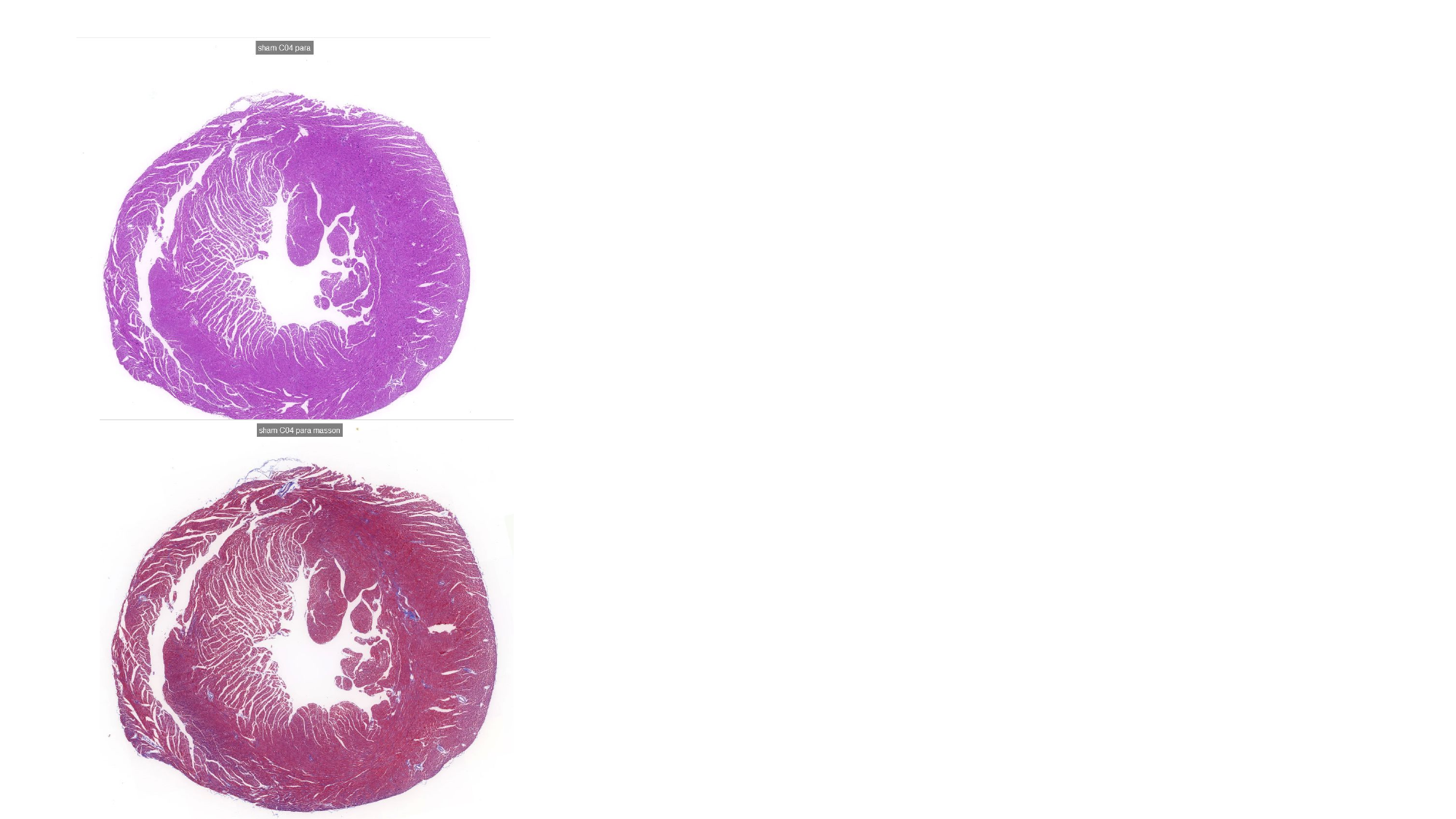

## Slide 6
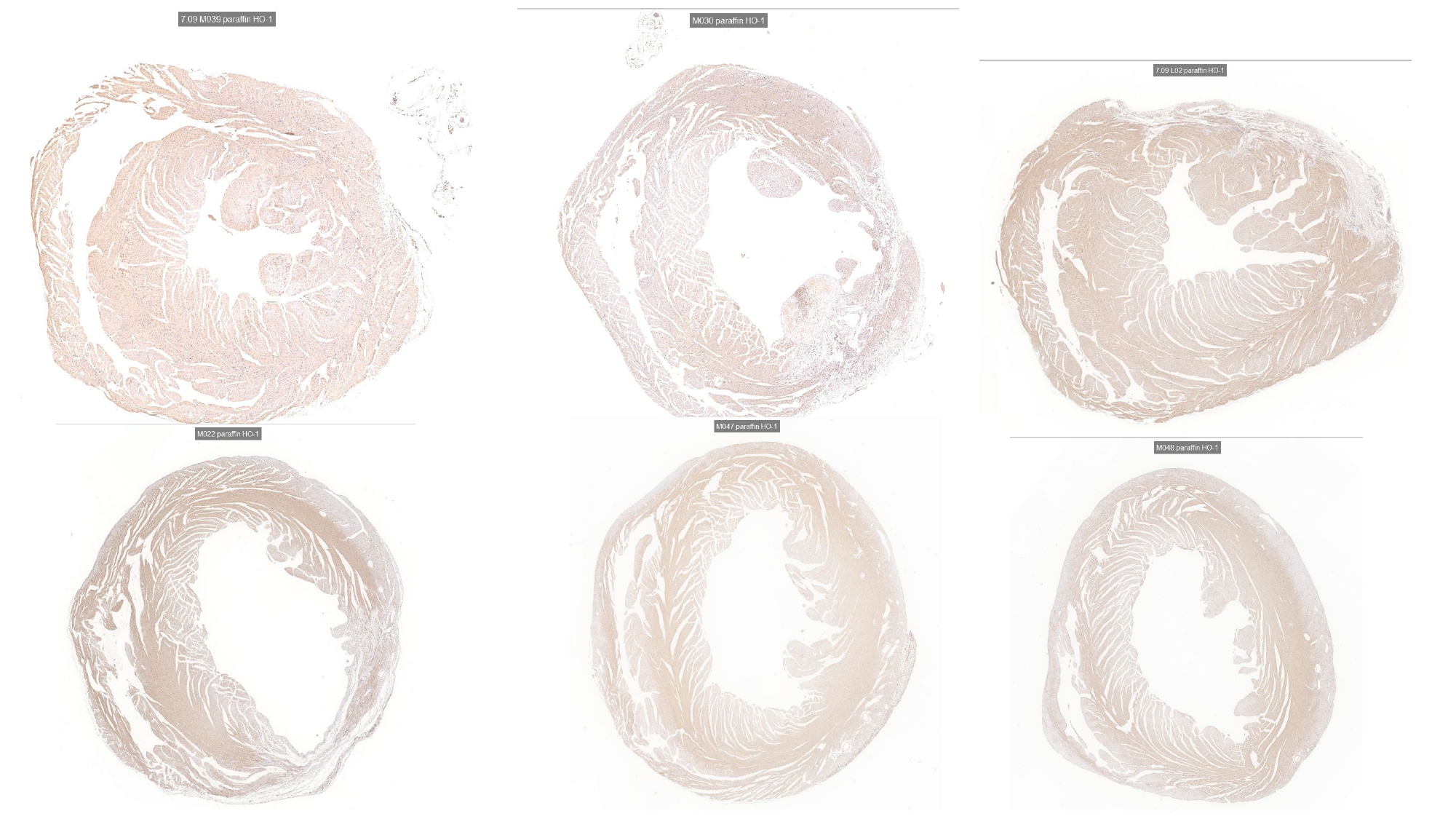

## Slide 7
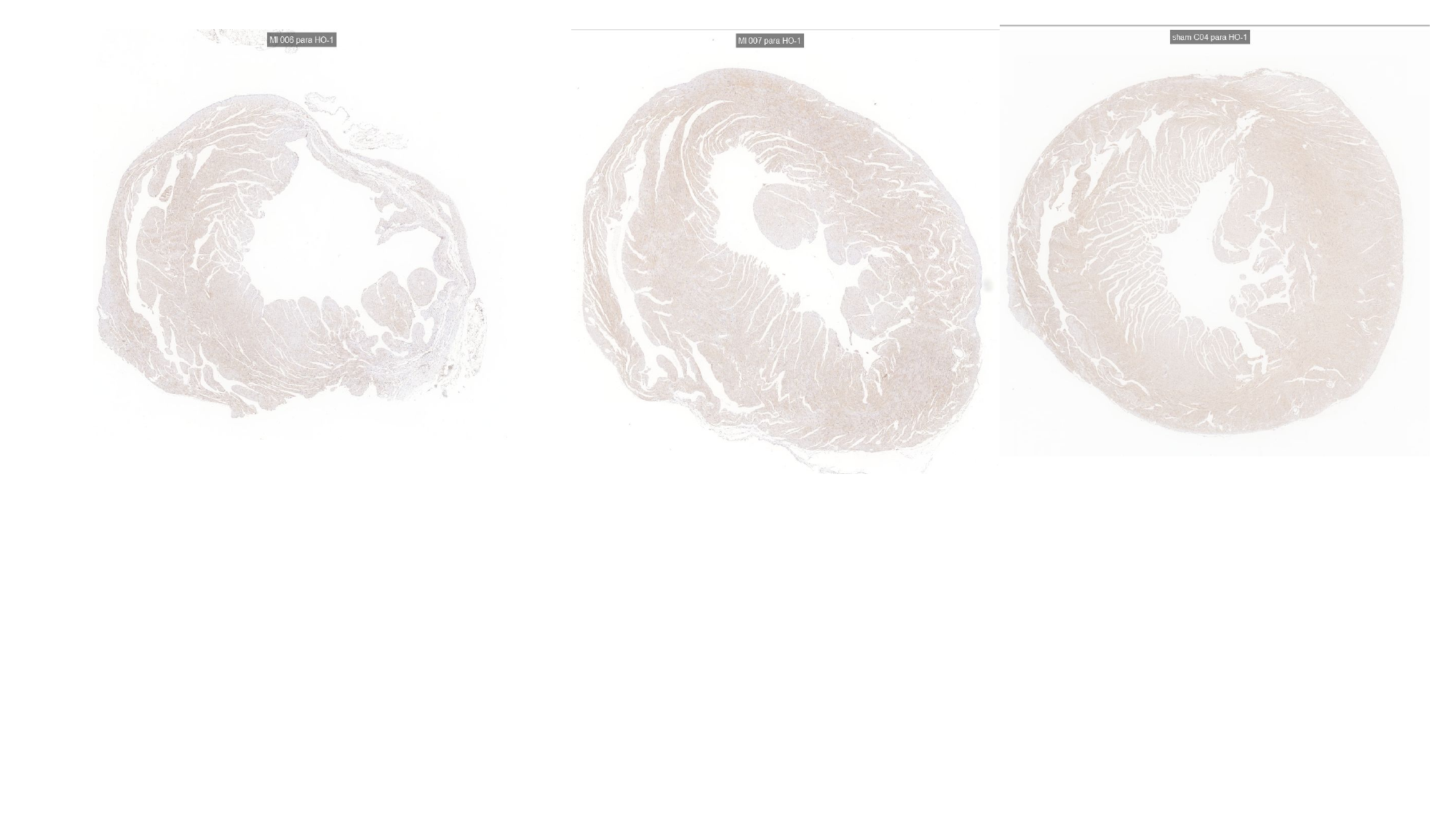

## Slide 8
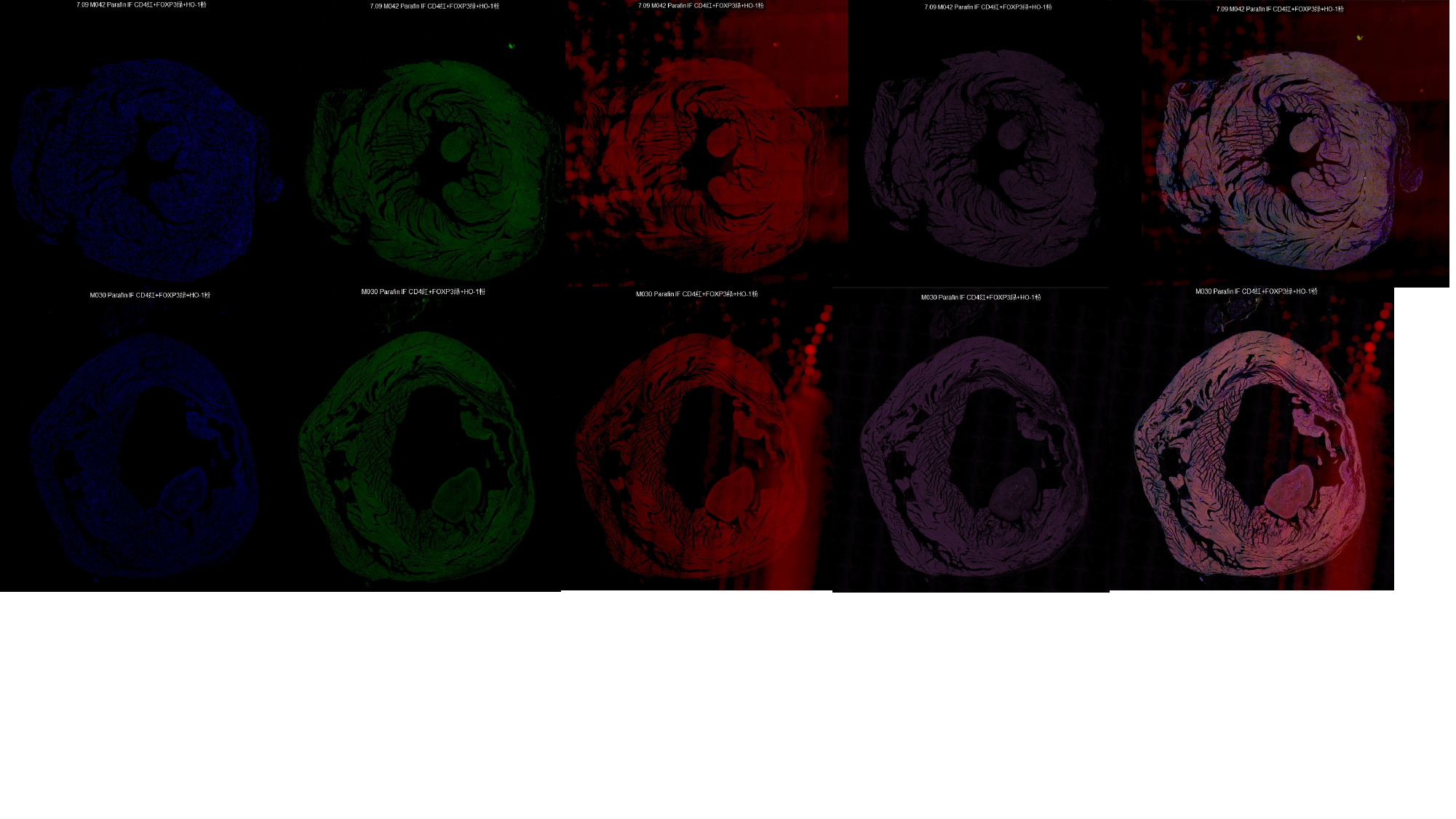

## Slide 9
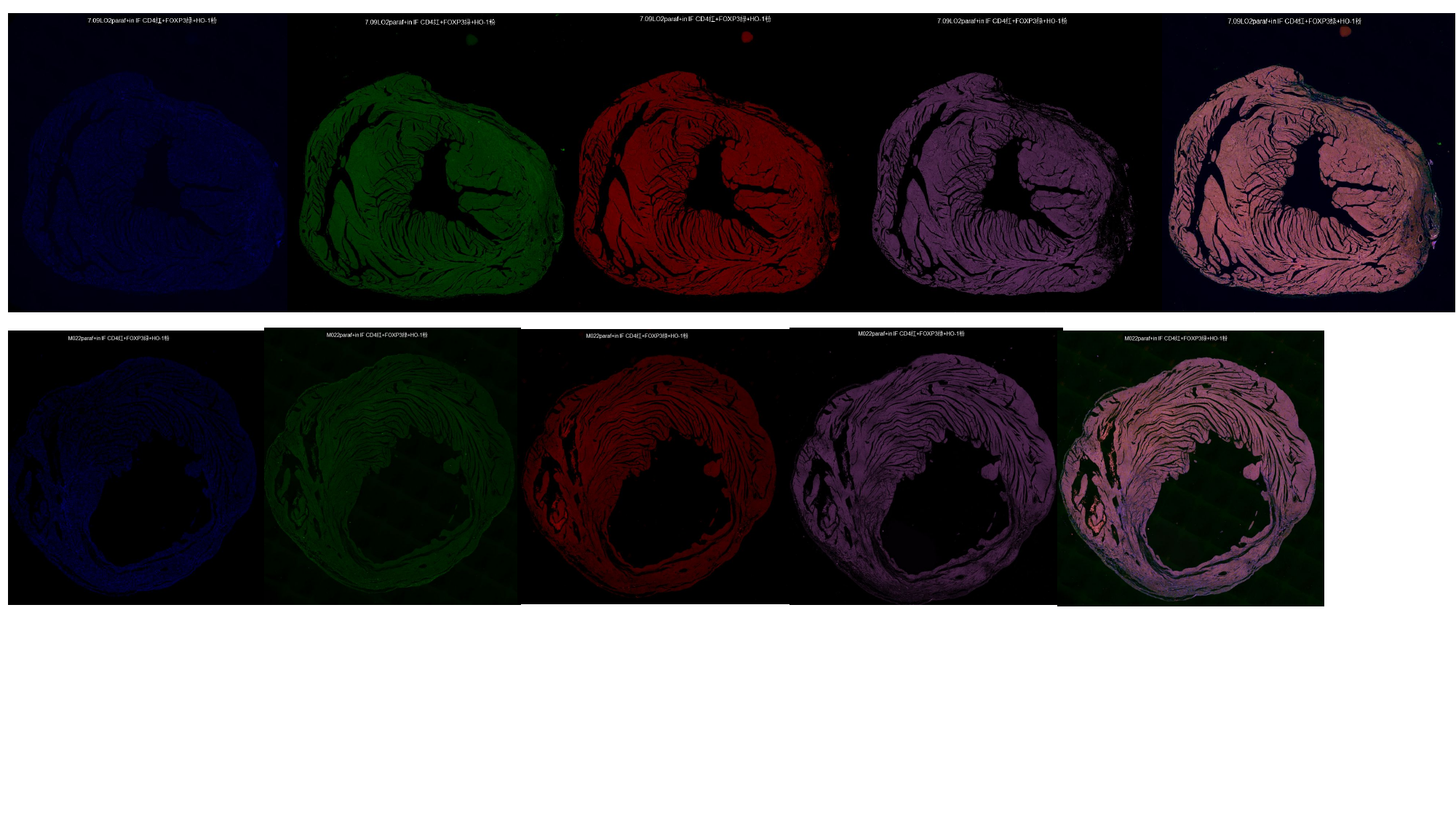

## Slide 10
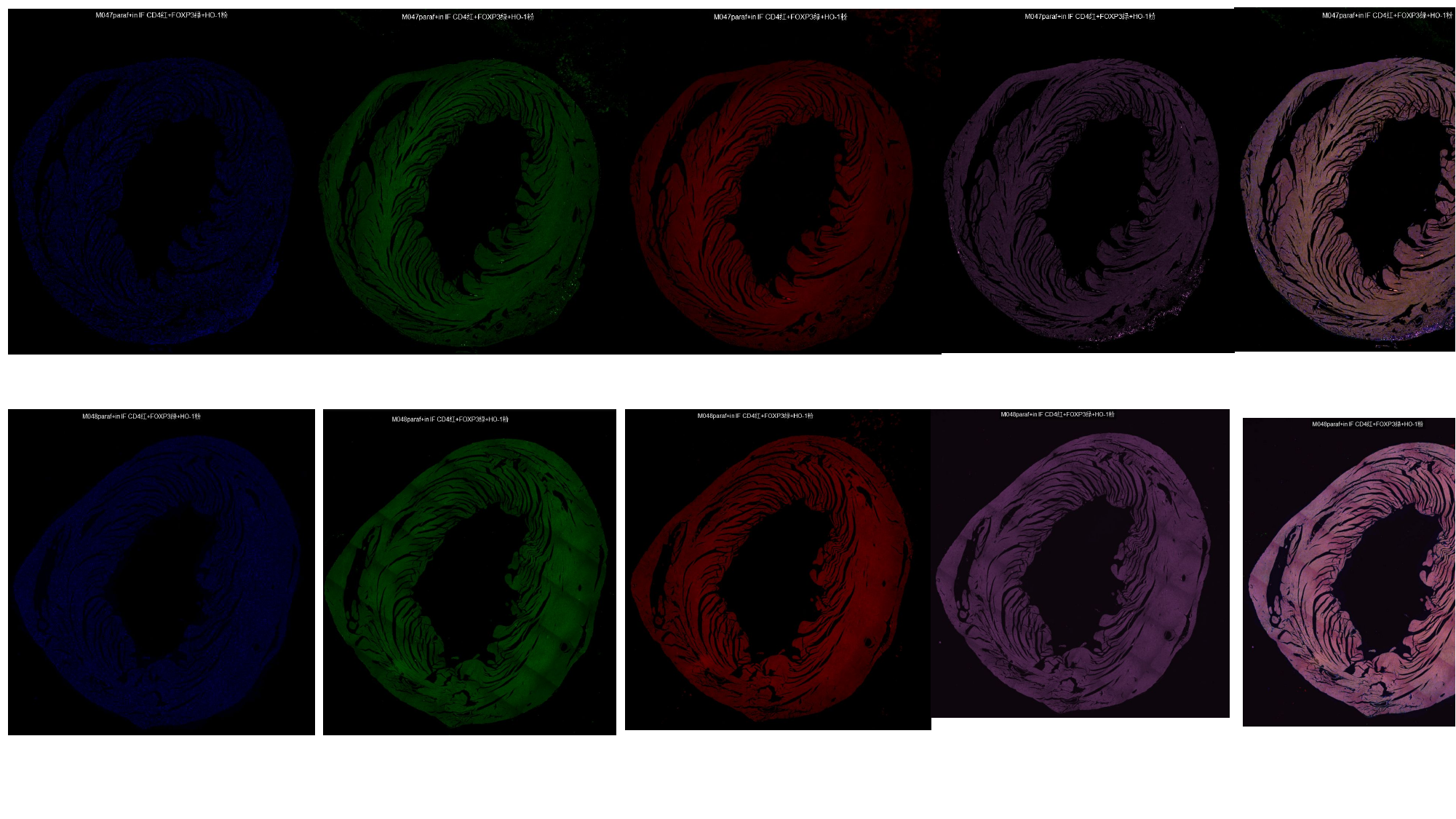

## Slide 11
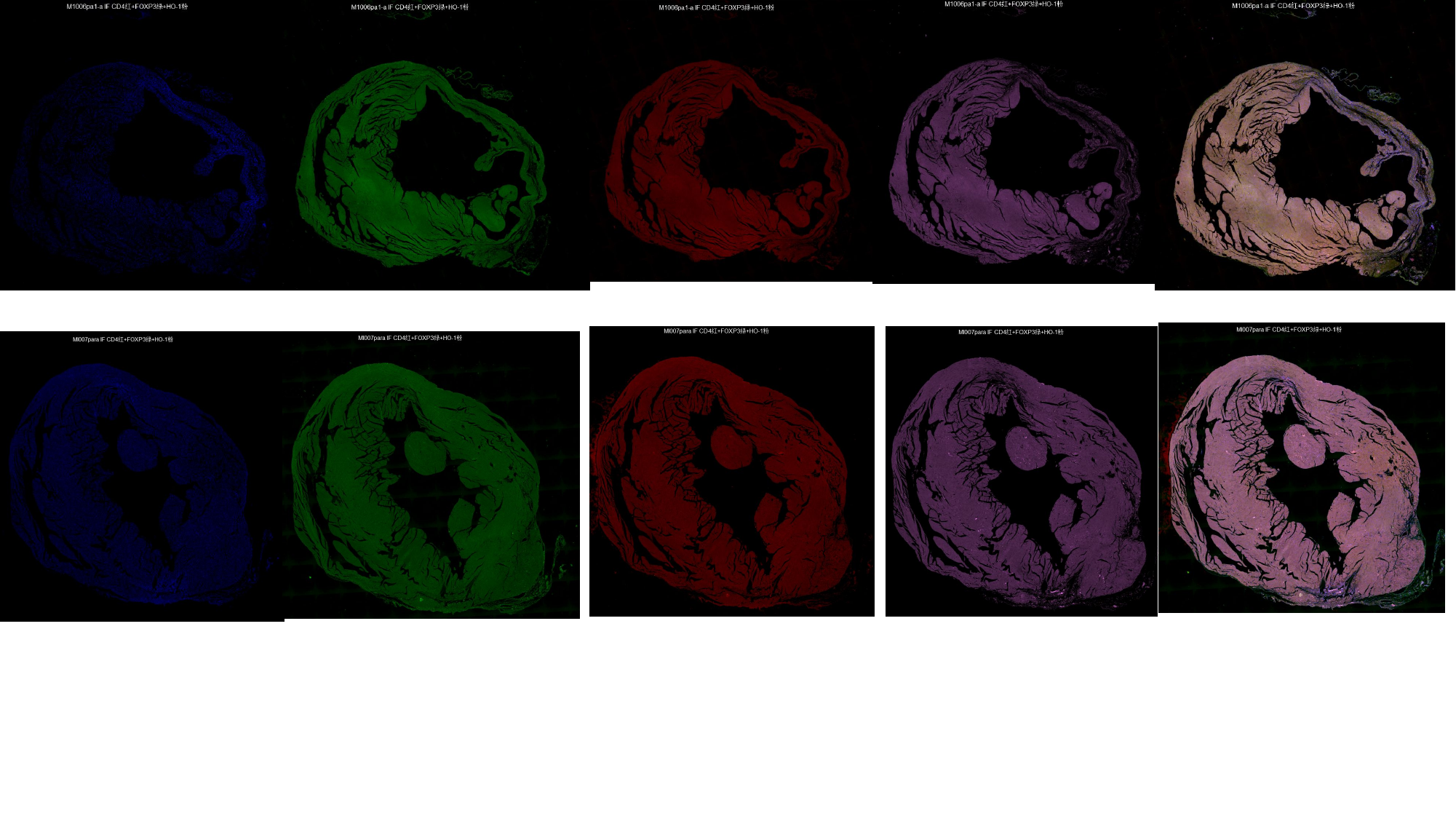

## Slide 12
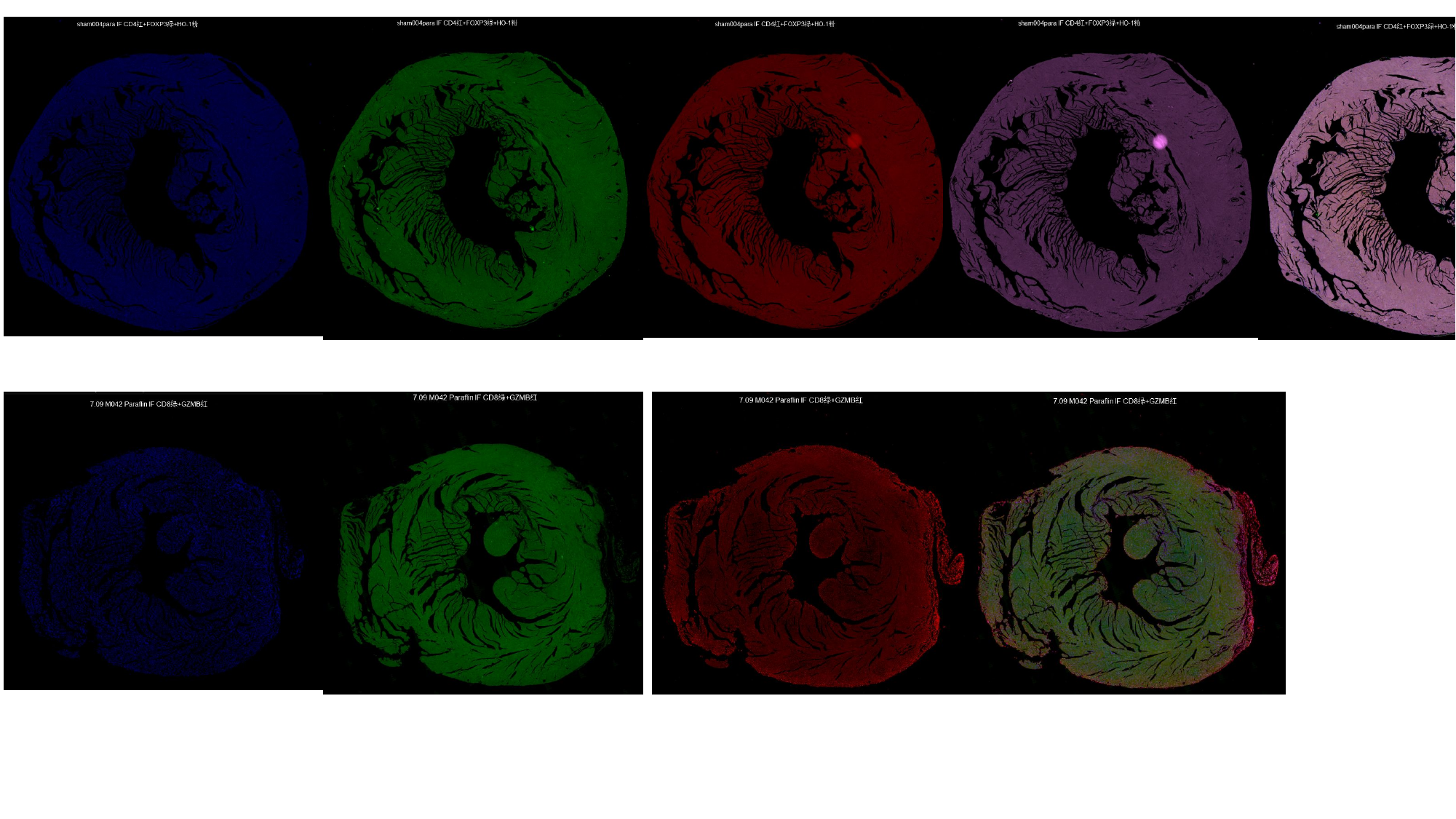

## Slide 13
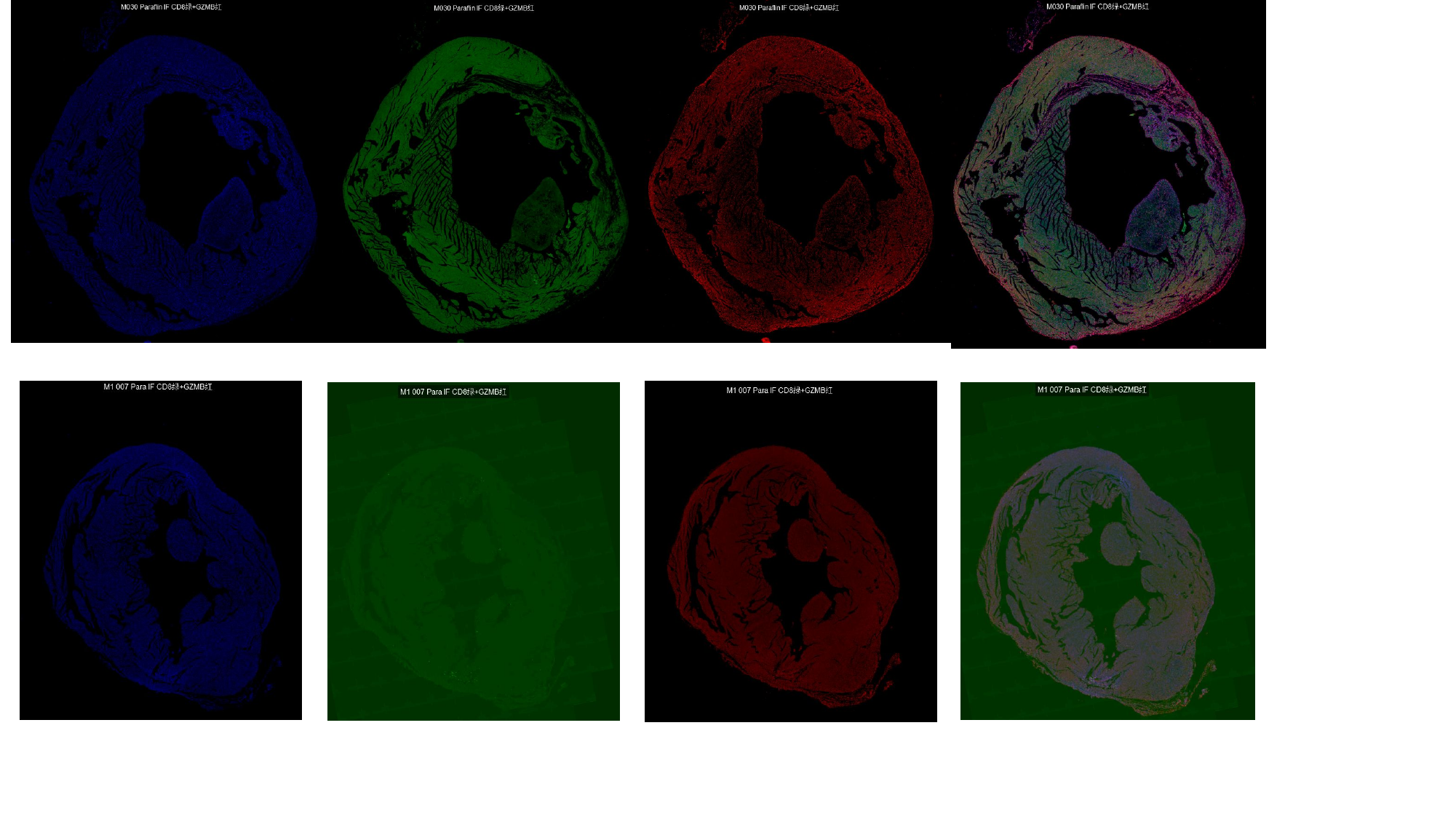

## Slide 14
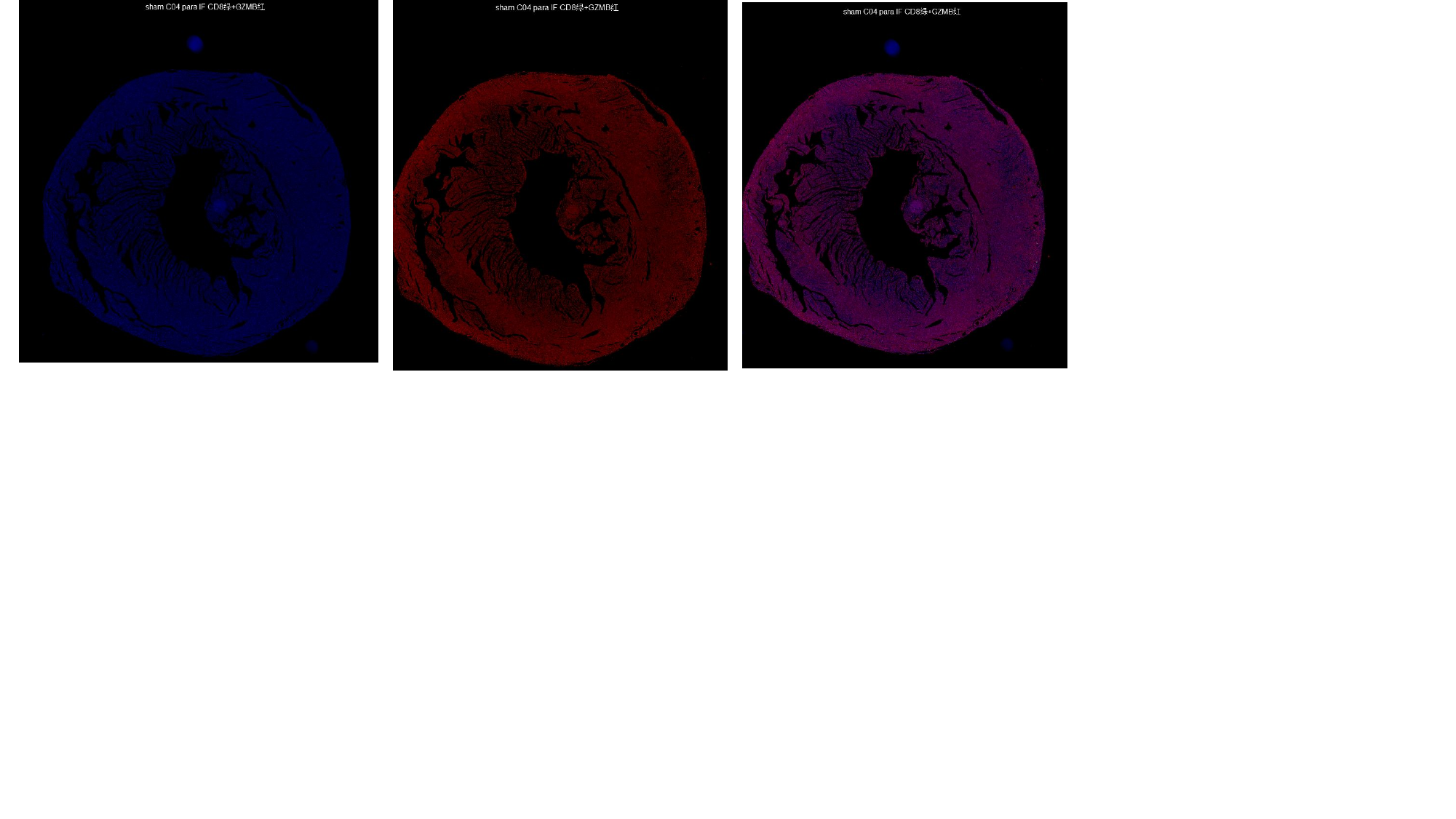

Supplement: Supplemental Material [file KBIE_A_2076453_SM5677.zip › supplementary/Raw data immuno.pptx]

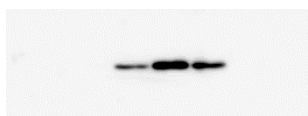

BAX

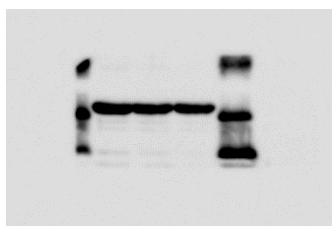

GAPDH

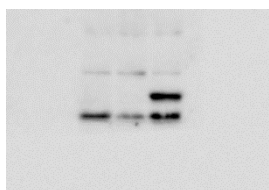

ho

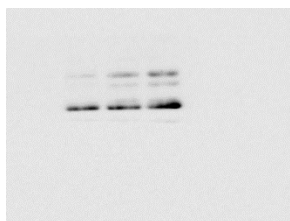

NRF2

Supplement: Supplemental Material [file KBIE_A_2076453_SM5677.zip › supplementary/raw data.pdf]
